# Confocal Laser Scanning Microscopy Evaluation of an Acellular Dermis Tissue Transplant (Epiflex®)

**DOI:** 10.1371/journal.pone.0045991

**Published:** 2012-10-02

**Authors:** Eric Dominic Roessner, Mario Vitacolonna, Peter Hohenberger

**Affiliations:** Division of Surgical Oncology and Thoracic Surgery, Department of Surgery, University Medical Centre Mannheim, Mannheim, Germany; University of Navarra, Spain

## Abstract

The structure of a biological scaffold is a major determinant of its biological characteristics and its interaction with cells. An acellular dermis tissue transplant must undergo a series of processing steps, to remove cells and genetic material and provide the sterility required for surgical use. During manufacturing and sterilization the structure and composition of tissue transplants may change.

The composition of the human cell-free dermis transplant Epiflex® was investigated with specific attention paid to its structure, matrix composition, cellular content and biomechanics.

We demonstrated that after processing, the structure of Epiflex remains almost unchanged with an intact collagen network and extracellular matrix (ECM) protein composition providing natural cell interactions. Although the ready to use transplant does contain some cellular and DNA debris, the processing procedure results in a total destruction of cells and active DNA which is a requirement for an immunologically inert and biologically safe substrate. Its biomechanical parameters do not change significantly during the processing.

## Introduction

### Extracellular Matrix as a bioactive Material for tissue reconstruction

Scaffolds in engineered tissues are used to mimic the ECM of native tissues. An ideal scaffold material should be stable, easy to use, biodegradable and non- toxic to cells. Furthermore the scaffold should closely resemble the body's extracellular matrix to facilitate optimal growth and differentiation of cells. The extracellular matrices that are available for medical purposes have different origins. They may be xenogenic, allogenic, autologous, semisynthetic or synthetic in nature, resulting in varying biological and mechanical properties [1]. Decellularized xenogenic or allogenic matrices, e.g. from split-thickness skin, fulfill most of the prerequisites of a natural scaffold [2]. Such biological scaffolds from decellularized tissues have been successfully applied in animal studies and clinically. [3]–[7]. However allogenic or xenogenic antigens can induce an inflammatory response or result in acute rejection of the implant [8], [9]. Therefore the decellularization of the acellular dermal matrix (ADM) has to be as complete as possible, without adversely affecting the composition, biological activity or the mechanical integrity of the ECM. Remaining non cellular antigens may induce a desirable constructive immune response.

One of the main problems in processing ADM is that valuable matrix constituents such as glycosaminoglycans may be removed, resulting in an alteration of the native integrity and architecture of the matrix. The mechanical properties of the matrix may be altered, influencing its behavior as a scaffold for cell seeding. The ECM acts as a superstructure with various structural proteins permeated by interstitial fluid constituents and soluble signaling molecules. This enables tissues to maintain their form while allowing for diverse host processes such as angiogenesis, cell migration, cell proliferation, inflammation and wound healing. The loss of these important adhesion molecules and fibers after processing may lead to a disturbance in the vascularization, migration and growth of cells after *in vivo* or *in vitro* repopulation [10]. It is therefore crucial when decellularizing the ECM to find a balance between the maximal removal of the antigenic cellular material and the retention of the native mechanical and biological properties of the constituent structural proteins such as collagen and fibronectin [11].

The purpose of this work was to examine an acellular dermis transplant (Epiflex®) for its composition and structure after completion of the processing procedure. The extracellular matrix was then further assessed for cellular and DNA content as well as biomechanical properties.

## Material and Methods

### Preparation of acellular dermis transplant

A detailed description of the techniques used in the development and processing of the acellular dermis transplant Epiflex® has been previously published [12]. The transplant is manufactured by the German Institute for Cell- and Tissue Replacement (DIZG), Berlin, Germany. The skin used for the manufacture of Epiflex® is recovered from screened consenting donors according to validated and approved methods. Skin pieces are recovered with a dermatome and stored at −40°C. Remnants of blood, fat and connective tissue are removed and damaged areas (necrosis, tears, holes) are excised and discarded. For decellularisation the skin pieces are soaked completely submersed in a sequence of hypertonic salt solutions over a period of 48 hours at room temperature in a shaking water bath. The epidermis is removed with forceps and discarded. The remaining dermal tissue is then placed in an aqueous dilute anionic detergent solution for 24 hours at RT and is then washed intensively. Decellularized dermis is placed in a pressure resistant vessel completely submerged in a Peracetic acid-based sterilisation solution, the vessel is sealed and evacuated by means of a vacuum pump and shaken vigorously for 4 hours. Residual sterilant is then removed by continuous automated washing with WFI until a downstream MerckoQuant^TM^ Peracetic acid test (VWR International GmbH Deutschland, Dresden, Germany) of the wash effluent delivers a result of <1 ppm Peracetic acid. After sterilisation the tissue is stored in sterile sealed trays and freeze-dried according to a proprietary protocol.

### Preparation of cryocuts

Normal skin and processed dermis were rehydrated for 30 Minutes in PBS, embedded in Tissue Freezing Medium (Tissue Tek®, Sakura), frozen at −80°C, and cryosectioned on dermis side at a thickness of 10 µm (skin) and 20 µm (ADM) using a Cryocut 1800 microtome.

### Autofluorescence

The structure and composition of the extracellular matrix was investigated by means of confocal laser scanning microscopy (Leica TCS SP2) based on collagen autofluorescence at 488 nm. A z-step of 0.2 µm was used to optically section the samples. Observations with 20x, 40x and 63x oil immersion lenses were performed. Data files were saved in tiff format and processed with ImageJ (v.1.42b) Software.

### Immunofluorescence

Cryosections were fixed in acetone, following PBS washing. Sections were then incubated with the primary antibody at RT in a humidified chamber. Fluorescence- conjugated secondary antibody with species specificity appropriate for each primary antibody were added, and incubated. The sections had an area of 1 cm×1 cm and were divided into 10 grid units for evaluation.

### Matrix Components

The following primary antibodies were used, each diluted 1∶100: rabbit anti-human collagen I (Rockland, USA), rabbit anti-human collagen II (Rockland, USA), rabbit anti-human collagen III (Abcam, UK), rabbit anti-human collagen IV (Rockland, USA), rabbit anti-human fibronectin (Abcam, UK), sheep anti-human hyaluronic acid (Biotrend, Germany), mouse anti-human laminin-5 (BD Bioscience, USA), rabbit anti-human laminin (Rockland, USA), mouse anti-human osteopontin (Santa Cruz Biotechnology, USA), mouse anti-human tenascin (NeoMarkers, USA), rabbit anti-human vitronectin (Biotrend, Germany), mouse anti-human thrombospondin-1 (Dianova, Germany). The following secondary antibodies were used, each diluted 1∶100: cy^5^-conjugated donkey anti-rabbit IgG (Jackson ImmunoResearch, USA), TexasRed-conjugated goat anti-mouse IgG (Jackson ImmunoResearch, USA), cy^5^-conjugated Strepavidin (Jackson ImmunoResearch, USA). For the staining procedure, the immunofluorescence protocol described above was used. Each Antibody was used on n = 10 samples.

### Assessment of Cellular Content

The following primary antibodies were used: mouse anti-human α-, β-, γ -catenin (BD Bioscience, USA) (1∶50), rabbit anti-human VEGFR-1 (Biotrend, Germany) (1∶100), rabbit anti-human VEGFR-2 (Biotrend, Germany) (1∶100), rabbit anti-human von-Willebrandt-Factor (Abcam, UK) (1∶100) and Tritc-conjugated Phalloidin (Sigma_Aldrich) (1 µg/ml). The following secondary antibodies were used, each diluted 1∶100: TexasRed-conjugated goat anti-mouse IgG (Jackson ImmunoResearch, USA), TexasRed-conjugated goat anti-rabbitIgG (Rockland, USA). For the staining procedure, the immunofluorescence protocol described above was used. To score the rare cellular content, we have divided the area of 1×1 cm from every slide in 4 quadrants. Each Antibody was used on n = 10 samples.

### DNA-Content

The following reagents were used: Propidium-iodide (PI) (0.5 µg/m, 2.5 µg/ml and 5 µg/ml), RNAse (1 mg/ml) and DNAse (1 mg/ml). Samples were treated with PI, DNAse+PI, RNAse+PI and DNAse+RNAse+PI, the incubation period of DNAse and RNAse was 30 minutes at RT. For PI staining, the immunofluorescence protocol described above was used. PI was used on n = 10 samples.

### DNA Quantification and Fragment Length Analysis

To quantify total DNA content, a modification of Gilbert's method [13] was used. Acellular dermis transplants from 8 donors (each in triplicate, n = 24) were cut into small strips and digested with 50 µl Proteinase K (1 mg/ml) in 500 µl PBS at 37°C for 72 h. Digested Proteins were then precipitated with Puregene Tissue Kit (Qiagen). Supernatants were purified with Isopropanol and 70% Ethanol, followed by centrifugation at 14000 rpm for 10 min and rehydration in 20 µl DNA-Hydration-Solution. DNA content was quantified using a NanoDrop™ Spectrophotometer (Peqlab). To determine DNA fragment size, samples were separated by electrophoresis on a 3% agarose gel containing ethidium bromide at 100 V for 30 min, and visualized with an ultraviolet transilluminator.

### Standardized Biomechanical Testing

Samples were rehydrated for 30 Minutes in PBS and punched out with a standardized die cutter according to German and international industrial standard (DIN 53455, ISO 527-1) [14]. All specimens had dimensions of 4.5×2 cm. The specimens were placed in a universal testing machine (Modell, Zwick/Roell, Germany; strain rate 50 mm/s) and subjected to uniaxial extension until failure. Load and displacement were recorded. Biomechanical analyses were performed on n = 8 samples for each of two specimen types; thin (0.3 – 0.8 mm) and thick (>0.8 mm).

### Statistical analysis

Data was imported into Microsoft Excel (Microsoft) for analysis of mean values and standard deviation. Paired *t*-tests were performed to evaluate significant differences (P≤0.05) between the two specimen types (thin and thick) using standard statistical analysis software (SPSS).

## Results

### Three-dimensional architecture

**Figure 1:**
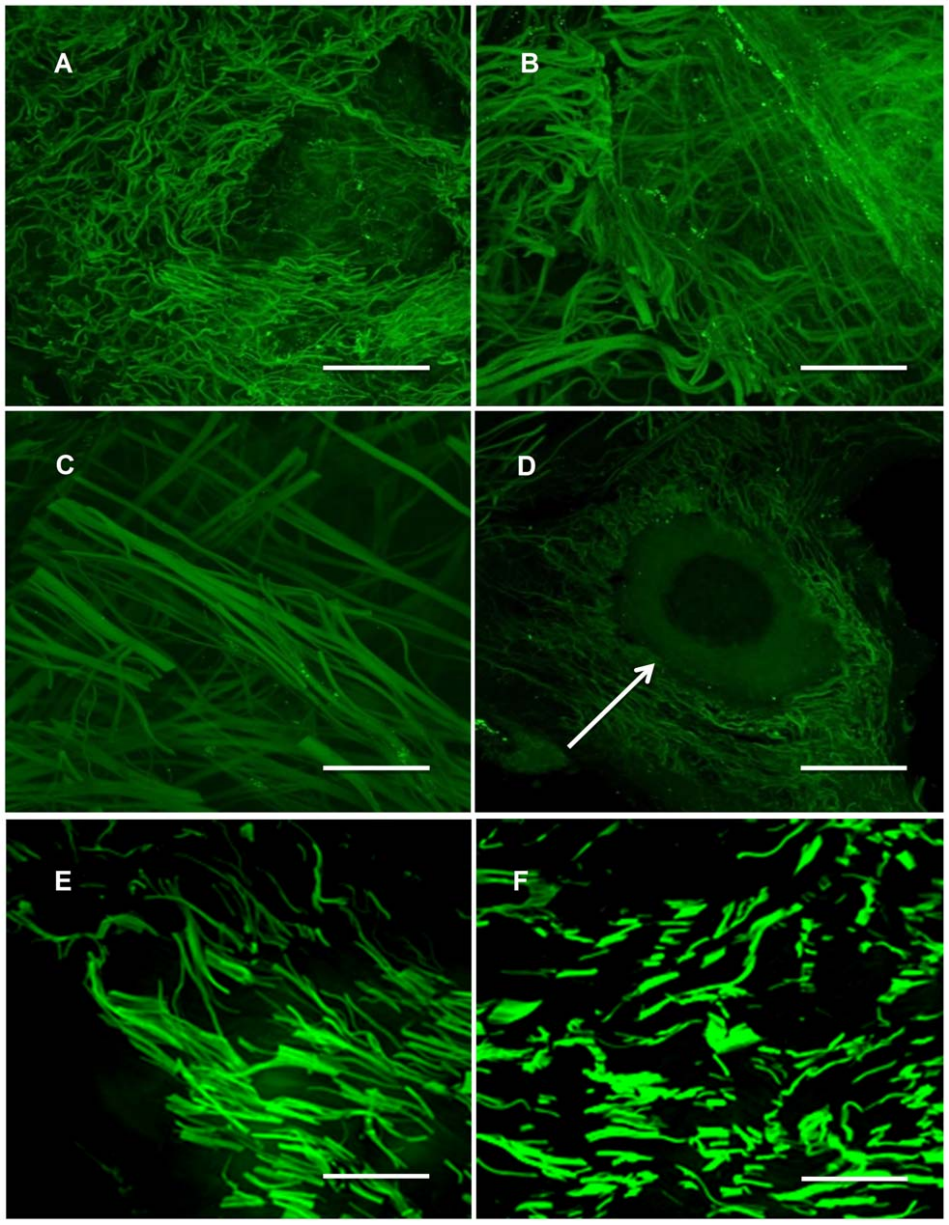
Confocal microscopy: (A-D) Confocal microscopy autofluorescence images of 20 µm ADM cryocuts, scale bars equal (A,C) 75 µm, (B,D) 50 µm; (D) the white arrow indicates a putative vessel channel; (E,F) autofluorescence images of 10 µm cryocuts of native human skin, scale bars equal (E,F) 75 µm.

In order to assess the configuration of the fibers and to investigate the matrix for presence of typical dermal structures such as vessel channels, we visualized 20 µm cryosections of the ADM by means of confocal laser microscopy based on the high autofluorescence of the tissue at 488 nm. All autofluorescence images showed the integrity of the natural structure of the fiber network with preserved gross structures including blood vessel canals (Figure 1D). The fibers are largely intact and have a dermis-typical size, density and distribution (Figure 1E,F). The appearance is partly non-homogeneous, for the most part consisting of parallel fiber bundles with some fine network structures (Figure 1B).

### Extracellular matrix components

**Figure 2:**
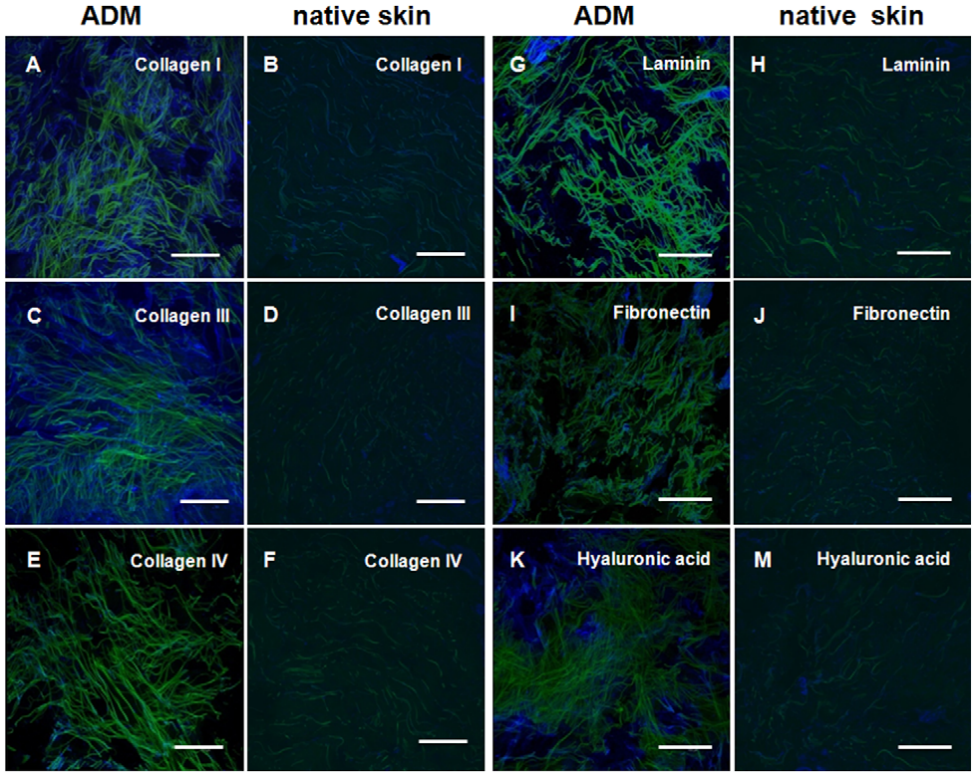
Confocal microscopy overlays of autofluorescence scans (green) and the respective anti- matrix antibody staining (blue); comparison of 20 µm cryocuts of ADM (A,C,E,G,I,K) and 10 µm cryocuts of native human skin (B,D,F,H,J,L); scale bars equal 150 µm. (**A,B**) anti- collagen I (**C,D**) anti- collagen III (**E,F**) anti- collagen IV (**G,H**) anti- laminin 1 (**I,J**) anti- fibronectin (**K,L**) anti- hyaluronic acid.

Collagen I, III, IV, fibronectin, laminin, vitronectin and hyaluronic acid were found to be present within the matrix, differing in their amounts and distribution (Table 1).To understand how the auto- fluorescent fibers are associated with the ECM proteins, we measured the auto- fluorescence of the fibers simultaneously with the ECM components, which were stained using specific antibodies against ECM components. The images in Figure 2 were then created by an overlaying technique. Green indicates auto- fluorescence whereas the ECM proteins are depicted in blue. For comparison with unprocessed tissue, we stained native skin with the same anti-ECM Antibodies (Figure 2B,D,F,H,J,M). Due to intact tissue containing cells, the fluorescence is more attenuated than in decellularized dermis. The immunostaining showed that the majority of the ADM consists of collagen I and III fibers (Figure 2A, B). Both fibrillar collagens showed an intense homogeneous staining. In normal tissue, type IV collagen (Figure 2C) is prominent in the basement membrane and in the dermal vascular bundles. The amount of type IV collagen seems to be less than the amounts of collagen I and III but was nevertheless present. Laminin-1 (Figure 2D), normally present in the basement membrane of unaltered tissues, could be found in moderate amounts. Fibronectin (Figure 2E), which appears in the ECM as free fiber as well as in association with collagens was detected in smaller amounts than collagen I and III. Hyaluronic acid, a large GAG normally involved in maintaining ECM hydration, growth factor binding and cell signaling [15] could be detected in abundant, homogeneously distributed quantities (Figure 2F). Small amounts of Vitronectin were found to be diffusely distributed through the ADM, whereas Collagen Type II and matricellular proteins like osteopontin, thrombospondine-1 and tenascin could not be detected by immunofluorescence staining.

**Table 1:** Summary of immunostaining with human antibodies against matrix components of the ADM; + means detectable by immunostaining, - means absence of any detectable signal.

Antibody	Results of staining
**Collagen I**	++
**Collagen II**	−
**Collagen III**	++
**Collagen IV**	+
**Fibronectin**	+
**Laminin-1**	+
**Laminin-5**	−
**Hyaluronic Acid**	++
**Vitronectin**	+
**Osteopondin**	−
**Thrombospondin-1**	−
**Tenascin**	−

### Assessment of DNA Content

**Figure 3:**
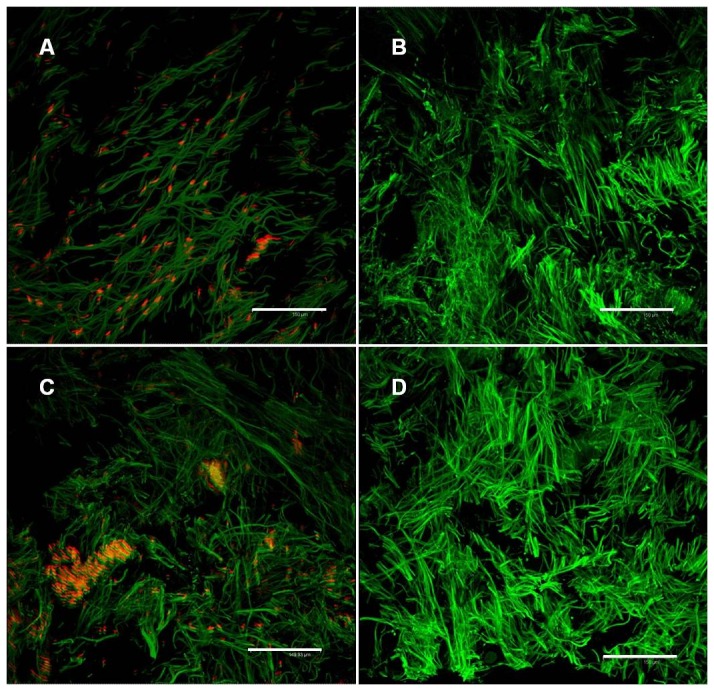
Confocal microscopy overlays of autofluorescence scans (green) and PI staining (orange), 20 µm cryocuts, scale bars equal 150 µm. (**A**) only PI (**B**) treatment with DNAse+PI (**C**) treatment with RNAse+PI (**D**) double treatment with DNAse+RNAse+PI

**Figure 4:**
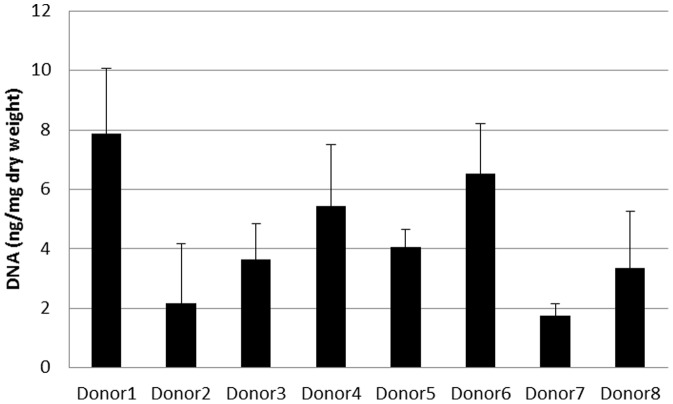
DNA content from 8 different donors.

**Figure 5:**
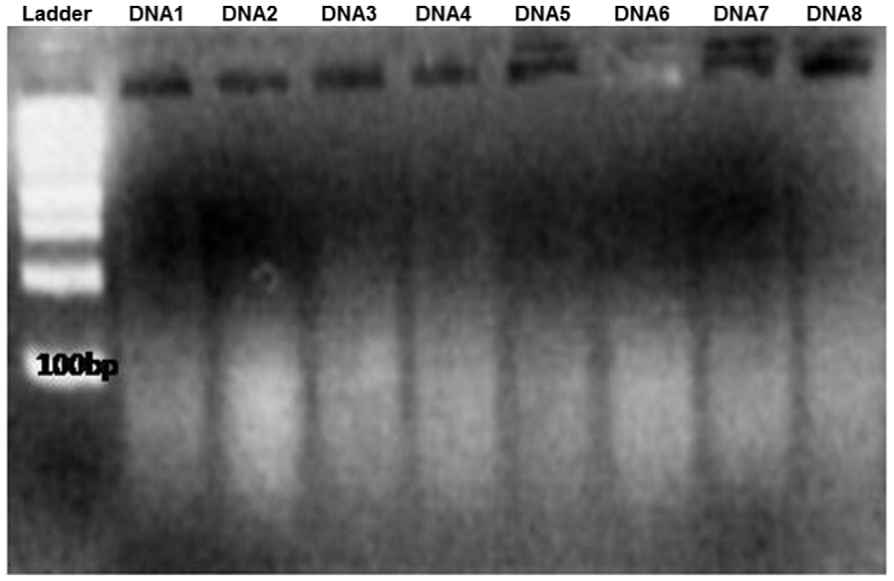
Gelelectrophoresis - DNA from 8 different donors, as DNA marker a 100bp DNA ladder was used.

The ADM contained measurable amounts of DNA as determined qualitatively by immunofluorescence using confocal microscopy with propidium- iodide as an intercalating agent (Figure 3A, red) and quantitatively by measurement with a NanoDrop™ Spectrophotometer (Figure 4). The values vary from 1.76±0.38 ng DNA/mg dry mass to 7.86±2.20 ng DNA/mg dry weight. To ensure that positive results were DNA, the samples were treated with DNAse and RNAse before immunofluorescence staining. After treatment with DNAse, no positive signals could be found (Figure 3B), whereas treatment with RNAse showed again positive staining (Figure 3C). No DNA was measurable with Nano Drop™ after DNAse treatment. Figure 3D shows incubation with DNAse and RNAse before staining. Gel electrophoresis of the samples showed that the majority of the DNA fragments were present primarily in the size range of 100bp and less (Figure 5).

### Assessment of Cellular Content

**Figure 6:**
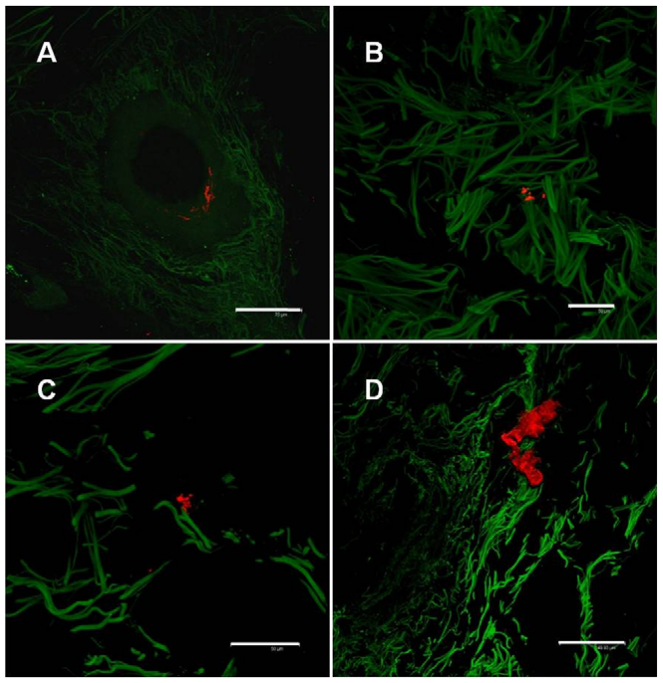
Confocal microscopy overlays of autofluorescence scans (green) and the respective positive cell surface and intracellular antibody (red) staining, 20 µm cryocuts, scale bars equal (A,B,C) 50 µm, (D) 150 µm. (**A**) Anti. α-Catenin (**B**) Anti- Cytokeratin (**C**) Anti- vWF (**D**) Anti- VEGFR1

In order to assess whether cells were present within the matrix, immunofluorescence staining with antibodies to α-, β-, γ- Catenin, VEGFR1, VEGFR2, vWF, Cytokeratin and Phalloidin was performed. The analyzed sections were negative for the most part and no intact cells were found. However, within 8 quadrants in 10 examined slides, isolated structures could be detected, which presumably represent cellular debris. These can be seen in Figure 6A-D.

### Biomechanical data

Mean and standard deviations (SD) are listed in Table 2. The breaking force of the thin ADM was 84.7±40.0N whereas that of the thick material was 161.63±80.41N. The *p*-Value of 0.0296 indicates a significantly higher strength of the thicker specimens. The maximum force varied from 98.7±36.5N (thin) to 196.7±66.7N (thick) with *P* = 0.0027. The elongation was 12.3±3.8 mm (thin) versus 20.4±8.1 mm (thick) with a *p*- Value of *P* = 0.0222, which indicates a significant difference between the two specimen types.

**Table 2:** Biomechanical results of the two available sizes *thin* and *thick* of the ADM; Values are presented as mean (SD), *P*- Value evaluate significances between the differences of properties of the different sizes.

Parameter	Thin (0.3–0.8 mm)	Thick (>0.8 mm)	*P* Value
**Breaking force (N)**	84.71±40.03	161.63±80.41	0.0296
**Maximum force (N)**	98.74±36.56	196.78±66.75	0.0027
**Elongation (mm)**	12.30±3.86	20.45±8.09	0.0222

## Discussion

### Architecture and matrix- components

Recent studies have shown that cells are not only influenced by the composition and strength of substrata, but also by their topography and porosity. The architecture of an ideal scaffold should provide generous void volume for vascularization, efficient metabolite and nutrient transport and support remodeling [16]. Furthermore, the porosity should be appropriate for the migration of cells into the matrix. For this reason, it is desirable to maintain the natural network structure. Epiflex® showed a relatively uniform distribution of fiber bundles with fine net-like structures and thick parallel fibers as found in unprocessed human skin (Figure 1 E,F). To assess the influence of processing on the biomechanical properties, we determined breaking force and elongation of the sample. Although various studies on native skin [17]–[20] and other matrices such as Allopatch or GraftJacket exist [21]–[23], the of lack of standards prevent direct comparisons to the literature values. We have used the ISO standard ISO EN 527-1 (DIN 53455), generally used to determine the tensile properties in plastic and which can also be applied to other materials with similar characteristics, since there is no ISO standard for decellularized skin. Through these defined experimental conditions, a comparison of the various matrices is made possible. The rupture of the samples occurred at 84.71±40.03N for the thin specimen and 161.63±80.41N for the thick specimen with respective elongations of 12.30±3.86 mm (thin) and 20.45±8.09 mm (thick). The preserved vessel channels we found may allow accelerated vascularization without the use of any pro-angiogenic growth factors [10]. An intact fiber structure, especially collagen I and III increases the mechanical stability and tensile strength, can modulate the biomechanical behavior of the scaffold and the acceptance of the implant *in vivo* [24]–[28].

Autofluorescence analysis alone does not enable a reliable distinction between the various components of tissue. Elastin may also fluoresce under the described conditions. In general, the intrinsic autofluorescence of cells and tissues is based on the excitation of endogenous fluorophores. However, considering the strong staining of collagen I and III (Figure 2A,B), it appears that the majority of the fibers within the ADM consist of these two proteins. The areas where is no correlation with the autofluorescence could perhaps be explained by the antibody reaction with tropocollagen molecules. Type IV collagen is non-fibrillar and can usually be found in the basal lamina. Laminins are also mostly found in the basal lamina and promote, in a manner similar to fibronectin, the adhesion of cells to collagen IV. Type IV collagen and laminin are the main components of basement membranes [29]. The presence of these two proteins within the ADM, albeit in small quantities, could be an indication of the preservation of the basal lamina or at least parts of it. While laminins influence the proliferation, growth and differentiation of cells [30], their most important role is in the development and maintenance of vascular structures [31]. The basal membrane also serves as an interface between epithelial and mesenchymal tissue by anchoring the intracellular keratin cytoskeleton of epithelial cells through hemodesmosomes to the basement membrane [32]. In this manner, a confluent epithelial cell layer on the luminal surface can be formed [33]. Fibronectin acts not only as an attachment protein but also plays a major role in tissue repair where it promotes the migration of fibroblasts during wound closure [34]. Hyaluronic acid, which belongs to the family of glycosaminoglycans, can bind large quantities of water, and supplements the viscoelastic properties of cartilage and joints. Furthermore, it plays an important role in binding growth factors and cytokines.

We have shown that the main components of the native extracellular matrix were present in different amounts within the ADM. The preservation of significant ECM components such as collagen type I, type III, type IV, fibronectin, laminin, vitronectin and hyaluronic acid in the Epiflex® represent an almost natural environment for cells and may enhance rapid recellularization and vascularization of the implant [10].

Currently available scaffolds such as the widely used small intestinal submucosa (SIS) or modified synthetic materials seem to be only compromise solutions. None of them are able to accurately mimic the natural ECM. The majority of studies in which the structural properties, ultrastructure and biological activity of a biomaterial have been assessed, have utilized SIS as the substratum. [35]. SIS is similar to Epiflex® in that Collagen I and III [36], GAGs such as hyaluronic acid [37] and adhesion molecules such as fibronectin and laminin [33], [38] are present. However SIS does not have an intact basement membrane [33]. Evidence of the presence of collagen IV and laminin within the ADM is not sufficient to deduce that an intact basement membrane is preserved. Other products such as xenogenic acellular dermis Strattice® are claimed to have a basal membrane. There has also been much research into developing a synthetic polymer which mimics the surface topography, mechanical properties and chemical composition of the natural ECM. These polymers have mostly been constructed from the combination of scaffolds constructed from electrospinning techniques [39]–[42] and subsequent modification with bioactive molecules such as laminin or collagen. The porosity and degradation rates of these synthetic materials may also be manipulated to allow for varying amounts of cellular tissue infiltration [43]. The resulting matrix, derived from modification of isolated proteins, however does not replicate the complexity of the natural ECM composition. In contrast Epiflex® seems to contain the combination of structural integrity and complex protein mixture which closely mimics the natural extracellular matrix.

The matricellular proteins including thrombospondin-1, thrombospondin-2, tenascin and osteopontinin in conjunction with the structural components of the ECM, regulate the intracellular signaling cascades of the cells and thus gene expression. This leads in turn to cell migration, differentiation and subsequent formation of complex tissue structures [44]. No matricellular proteins were detected by immunostaining. This may be because these relatively labile molecules were detached or degraded during processing or because the amounts present were so small that more sensitive methods such as ELISA would be required to detect them.

### DNA content and freedom from cells

Small quantities of residual DNA could be detected in Epiflex® by confocal microscopy via staining with propidium- iodide. A previous RT-PCR investigation of Epiflex for the GAPDH housekeeping gene has been described elsewhere [45]. In 8 out of 9 tested batches, the samples had no detectable copies of GAPDH as measured by RT–PCR. In 1 out of 9 samples, a weak signal at the detection threshold was recorded that may be attributable to a residual level of genetic material of ≤2 copies per mg tissue [45]. Therefore, it may be postulated that the residual DNA detected in the present study consists of non-transcribable DNA fragments. Moreover, the analysis of fragment length by gel electrophoresis shows that the DNA present consisted of fragments of less than 100bp length. This length of DNA is too short for a functional gene that can induce effects [13], [46]. Several commercially available allogenic and xenogenic materials, such as Restore™ (porcine small intestinal submucosa [SIS]), Graft Jacket™ (human dermis) or TissueMend™ (bovine dermis) also contain small amounts of DNA [13], [47]. Despite the presence of DNA and cellular debris in many of these commercially available scaffolds [13], these devices have successfully been used in numerous clinical studies [48]–[54]. Restore^TM^ SIS or Surgisis™ SIS (Cook Biotech) are examples of acellular xenograft materials that have official approval for clinical use. Some of these have been in clinical use for over a decade with no serious complications arising from the presence of DNA- remnants in their structure [35], [36], [55], [56]. The minimal amounts of remnant DNA and cell debris however, do not appear to play a role in these reactions [46]. And although the role of xenogeneic epitopes such as the galactose-α-(1,3)-galactose terminal carbohydrate epitopes (α-Gal) are currently controversially discussed in the rejection of xenogeneic graft materials [57], [58], [59], [60], [61], the use of a human implant can eliminate concerns about these species- specific Gal- and non-Gal glycoantigens [62], [63]

The immunofluorescence signals, which were observed in our CLSM investigation of Epiflex®, may be indicative of cellular debris. The level of cellular debris present in the Epiflex® is minimal, and is unlikely to result in any adverse effects. Whilst a total elimination of cellular debris and DNA fragments is desirable, this must be balanced against the structural and compositional consequences of the harsh methods that would be required to eliminate even the smallest quantities of cellular debris and DNA fragments.

Several other commercial human decellularized ECM matrices with similar properties and compositions have been described. These include: AlloDerm (Lifecell; skin), AlloPatch® (MTF; fascia lata), Axis™dermis (Mentor; dermis), Bard® Dermal Allograft (Bard; dermis), Graft Jacket® (Wright Medical Tech; skin) and Suspend™ (Mentor; fascia lata) [2]. However, it should be noted, that none of these materials are approved for use in Europe. Epiflex® is until today the only human ECM that is approved as a medical product in Germany. Such a “drug” approval requires more stringent licensing and control procedures, and it could be argued that increased patient safety is a consequence [12].

In summary, the present study shows that the processing of Epiflex® does not cause significant damage to the structure of the skin transplant and that relevant ECM proteins are retained. We therefore conclude that Epiflex ® is a favorable substrate.
